# The Italian Network for Monitoring Medication Use During Pregnancy (MoM-Net): Experience and Perspectives

**DOI:** 10.3389/fphar.2021.699062

**Published:** 2021-06-23

**Authors:** Valeria Belleudi, Filomena Fortinguerra, Francesca R. Poggi, Serena Perna, Renata Bortolus, Serena Donati, Antonio Clavenna, Anna Locatelli, Marina Davoli, Antonio Addis, Francesco Trotta

**Affiliations:** ^1^ Department of Epidemiology, Lazio Regional Health Service, Rome, Italy; ^2^ Italian Medicines Agency (AIFA), Rome, Italy; ^3^ Directorate General for Preventive Health - Office 9, Ministry of Health, Rome, Italy; ^4^ National Centre for Disease Prevention and Health Promotion, Istituto Superiore di Sanità - Italian National Institute of Health, Rome, Italy; ^5^ Laboratory for Mother and Child Health, Department of Public Health - Istituto di Ricerche Farmacologiche Mario Negri IRCSS, Milan, Italy; ^6^ Department of Obstetrics and Gynecology, University of Milano-Bicocca, Monza, Italy

**Keywords:** medication use, pregnancy, network, monitoring, appropriateness

## Abstract

There is an acute need for research to acquire high-quality information on the use of medicines in pregnancy, both in terms of appropriateness and safety. For this purpose, the Italian Medicines Agency established a Network for Monitoring Medication use in pregnancy (MoM-Net) through the conduction of population-based studies using administrative data available at regional level. This paper aimed to describe the experiences and challenges within the network. MoM-Net currently involves eight regions and several experts from public and academic institutions. The first study conducted aimed to identify drug use before, during and after pregnancy investigating specific therapeutic categories, analysing regional variability and monitoring drug use in specific subpopulations (i.e. foreign women/multiple pregnancies). Aggregated demographic, clinical, and prescription data were analysed using a distributed network approach based on common data model. The study population included all women delivering during 2016–2018 in the participating regions (*n* = 449,012), and corresponding to 59% of deliveries in Italy. Seventy-three per cent of the cohort had at least one drug prescription during pregnancy, compared to 57% before and 59% after pregnancy. In general, a good adherence to guidelines for pregnant women was found although some drug categories at risk of inappropriateness, such as progestins and antibiotics, were prescribed. A strong variability in the use of drugs among regions and in specific subpopulations was observed. The MoM-Net represents a valuable surveillance system on the use of medicines in pregnancy, available to monitor drug categories at high risk of inappropriateness and to investigate health needs in specific regions or subpopulations.

## Introduction

Medication use during pregnancy is a common event worldwide, which is rising in recent years. It is estimated that the prevalence of pregnant women receiving at least one drug ranges from less than 30% to over 90%, depending on the country and type of medication considered (AuthorAnonymous, 1992; Daw et al., 2011; Lupattelli et al., 2014; Haas et al., 2018).

During pregnancy, medicines can be taken for a wide range of acute or chronic clinical conditions. Treatment options should be evidence-based and favour the safest and most appropriate medicine for both, mother and foetus. (Dathe and Schaefer, 2019) Unfortunately, the risk/benefit profile of medication use in human pregnancy remains limited due to the frequent exclusion of pregnant women from clinical trials (Shields and Lyerly, 2013; Sheffield et al., 2014; Scaffidi et al., 2017). Thus, healthcare professionals and pregnant women, included those with chronic or long-term diseases, are often asked to make critical decisions about pharmacological treatments during pregnancy in absence of a clear clinical evidence. An accurate assessment of the risk-benefit profile between drug benefits for pregnant women and risks of adverse teratogenic, foetotoxic or developmental effects to the foetus is therefore strongly recommended (Westin et al., 2018; Stock and Norman, 2019).

In this context, there is an acute need for research to acquire high-quality information on medication use during pregnancy, both in terms of effectiveness and safety in order to inform health professionals and promote regulatory interventions. The real-world data, routinely collected by health information systems, may support these challenging needs (EUROmediCAT, 2015; Margulis and Andrews, 2017).

In recent years several multi-database initiatives, at national and international level, have been set up to secure surveillance of medication use in pregnancy (EUROmediCAT, 2015; Andrade et al., 2016; Bérard et al., 2019). The primary aim of these projects is to investigate the safety profile of specific drugs during pregnancy. Aspects related to pregnancy, such as the impact of pregnancy status on pre-existing chronic therapies, the appropriateness of drug use in pregnancy as well as the related compliance to clinical guidelines are poorly investigated (Tyer-Viola and Lopez, 2014; Kersten et al., 2014; D'Amore et al., 2015). From a public health perspective, it would be important to track the therapeutic pathways among pregnant women who live with a chronic disease and analyze potential differences in drug therapy patterns among specific vulnerable subpopulations of pregnant women with different chronic illnesses or poor health behaviours before pregnancy.

In Italy, population-based studies on drug use in pregnancy are few (Gagne et al., 2008; Ventura et al., 2018), dated and often coordinated at subnational level due to the lacking of a national observatory for monitoring drug prescriptive patterns over time.

Researchers call for long-term stable networks in order to provide rapid answers to urgent public health questions, particularly in special populations where information on drug safety and appropriate use may be lacking or very limited (Sultana et al., 2020).

In this perspective, the Italian Medicines Agency (AIFA) has recently promoted the creation of a network, including eight Italian regions and several experts from both public and academic Italian institutions, called MoM-Net (Monitoring Medication Use During Pregnancy Network), focusing on monitoring medication use in pregnancy, through the integration of different regional health databases.

The first project developed by the network aimed to evaluate the medication use before, during and after pregnancy in Italy by investigating: 1) drug prescription patterns in specific therapeutic areas; 2) regional variability in the use of medicines; 3) drug use in specific subpopulations, such as foreign women and women with multiple pregnancies.

The aim of this paper is to describe the framework of the project, study methods, the overall main results and future network’s perspectives.

## Project Management Framework

### Data Sources

A multi-database cross-sectional population study using a Common Data Model (CDM) was performed. The data sources used to identify the study population were:

• The Regional Birth Registry (*Certificato di Assistenza al Parto*, *CeDAP*), including information on socio-demographic (age, nationality, level of education, occupational status), characteristics of the mother and pregnancy (medically-assisted procreation, reproductive history, invasive prenatal test performed, gestational age, parity, c-section) and information on the newborn (length, weight, head circumference, 5-min Apgar-score, stillbirth, congenital anomalies);
• The Demographic Database, an administrative database collecting information on residents registered in the regional healthcare system;
• The Drug prescription database, including information on regional prescriptions reimbursed by the Italian National Healthcare Service (NHS), such as date of dispensing, number of packages, active ingredients and brand.

These data sources are linkable through a unique anonymous personal identification code at regional level.

**FIGURE 1:**
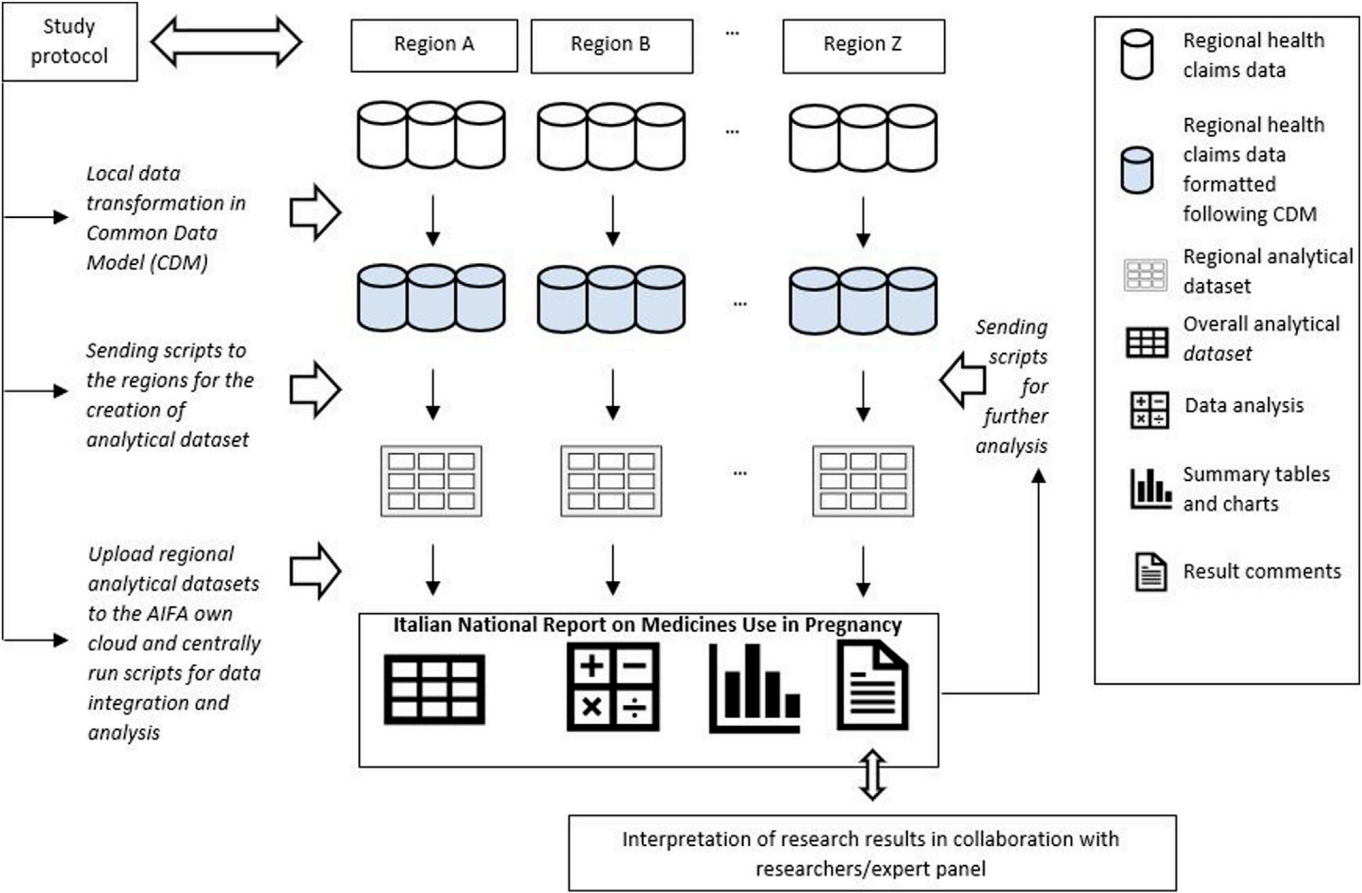
Analytical approach used to execute observational studies within the network MoM-Net.

The analytical approach used to perform the study consisted in a CDM designed by the Lazio region that led the data analyses (Gini et al., 2020; Schneeweiss et al., 2020). Through this approach, each participating region was required to translate the original regional administrative data into a predefined common data structure in order to standardize variable name, formats and modalities and to generate regional datasets univocally formatted and, at the same time, to allow the leading region to produce centrally script for manage and pre-processed locally data. Specifically, a common library of analytic routines was established and shared within the network. The local analytical datasets obtained after script execution were saved in a dedicated cloud and quality checks were performed. These datasets contained only the information strictly necessary to conduct the pre-planned analyses, without sensitive data which were previously anonymised at regional level by the data owner, in compliance with current Italian legislation on privacy [Figure 1].

The MoM-Net group includes epidemiologists, pharmacologists, analysts, biostatisticians and a panel of physicians and researchers with expertise in the field of maternal-fetal medicine. The main study results have been shared and discussed by the multidisciplinary group in order to provide accurate interpretation of data analyses.

### Analyses

The described analytical approach allowed to select all women aged 15–49 years living in eight Italian regions (Lombardy, Veneto, Emilia Romagna, Tuscany, Umbria, Lazio, Puglia, Sardinia) and giving birth in hospital between April 1, 2016 and March 31, 2018.

For each delivery, the date of pregnancy onset was estimated according to the gestational age at delivery, and three periods were identified as follows: 3 trimesters before, 3 trimesters during and 3 trimesters after pregnancy.

The prevalence of drug use was defined as the percentage of women with at least one drug prescription during the period considered (one of the three trimesters).

The overall prevalence of medication use before, during and after pregnancy was analysed focusing on prescribed active ingredients with a prevalence of use during pregnancy greater than or equal to 1%. The ranking of the top ten active ingredients by age group was, also, described.

Moreover, specific therapeutic categories were analysed taking into account three types of medications:

• medications prescribed for pregnancy (e.g. anti-anemic preparations, vitamins and sex hormones)
• medications prescribed for acute conditions (e.g. antibiotics for systemic use)
• medications prescribed for pre-existing chronic conditions (e.g. antiepileptic) or conditions occurring for the first-time during pregnancy (e.g., antihypertensives, antidiabetics, thyroid preparations, psychotropics).

For each therapeutic category and each trimester, the overall prevalence of drug use and the prevalence by woman’s age and drug subgroups were provided and showed using bar charts. According to the different therapeutic categories, the prevalence of drug use was investigated by maternal socio-demographic characteristics, reproductive history, pregnancy information and type of user, defined as “prevalent” when the drug was prescribed before conception or “incident” in case of new prescription during pregnancy.

**FIGURE 2:**
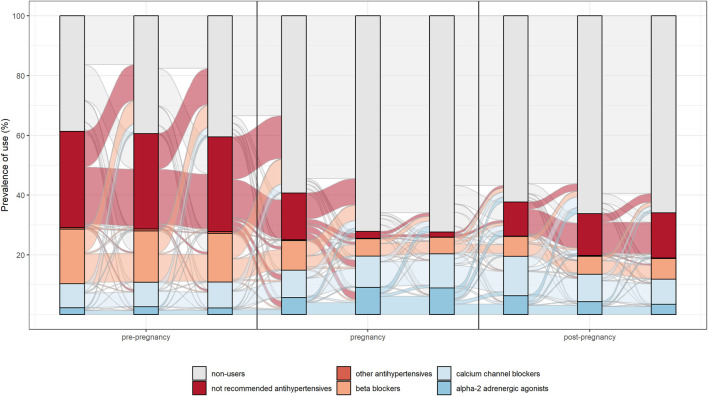
Sankey diagram used to describe the pattern of drug use in different trimesters of pregnancy: the example of antihypertensive in women with prevalent use during pregnancy.

**FIGURE 3:**
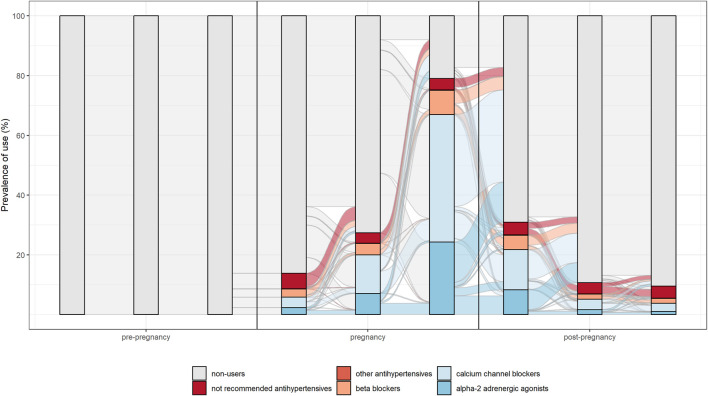
Sankey diagram used to describe the pattern of drug use in different trimesters of pregnancy: the example of antihypertensive in women with incident use in pregnancy.

The switch between different drug’s subgroups within the same therapeutic category was analysed. In particular, patterns of drug use in the different pregnancy trimesters were represented through the Sankey Diagram for all medications used in chronic diseases. This is a flow diagram in which the width of the arrows is proportional to the flow rate and it could be very useful in this context to report data on drug use in pregnancy. In fact, this visualisation technique allowed to display the percentage of women who interrupted treatments or switched from not recommended drugs to treatments of choice in pregnancy [Figures 2, 3 show an example of Sankey Diagram for antihypertensive medications].

Through an in-depth analysis, the combined use of different therapeutic categories in the same trimester or period (before, during and after pregnancy) was investigated.

The overall and specific prevalence of drug prescriptions by region and population subgroups, i.e. foreign women and women with multiple births, were calculated.

The variability of drug prescriptions among regions, although influenced both by different prescribing habits of physicians and disease epidemiology, depends on demographic characteristics of the study population. Therefore, in order to optimize the comparability between regions, the age-adjusted prevalence for the main therapeutic categories prescribed in each participating region was calculated Statistical analyses were performed using SAS and R.

## Main Results

Findings from the first study conducted by the MoM-Net are reported in the Italian National Report on medicines used in pregnancy, published in 2020 (The Medicines Utilisation Monitoring Centre, 2020). The data describe a cohort of about 450,000 women who gave birth in 2016–2018 in the eight participating regions, corresponding to 59% of Italian deliveries. The analysis shows that 73.1% of women who gave birth received at least one prescription during pregnancy, compared to 57.1% in the three trimesters before pregnancy and 59.3% in the three trimesters after childbirth (for details see Supplementary Table S1). The percentage of women receiving at least one drug prescription during pregnancy increased with maternal age, in line with results of other European cohort studies (Daw et al., 2011; Lupattelli et al., 2014; Network Italiano Promozione Acido Folico per la Prevenzione Primaria di Difetti Congeniti, 2015; Haas et al., 2018; Bérard et al., 2019).

Overall, study results show drug prescription patterns in line with evidence-based guidelines. In particular, for antihypertensive and antidiabetics a switch from not recommended subgroups of medications (such as oral hypoglycaemic medication, angiotensin-converting enzyme inhibitors and angiotensin receptor blocking agents) to treatments of choice (such as insulins, calcium channel blocker and centrally acting antiadrenergic) during pregnancy was observed.

However, for some drug categories a possible inappropriate use was identified. Specifically, it was found a peri-conceptional folic acid use, reimbursed by the NHS, lower than levels recommended by national and international clinical guidelines (The American College of Obstetrician and Gynecologists-ACOG, 2015; Wilson et al., 2015), a potentially inappropriate use of progestins during the first trimester, where the high proportion of users was probably linked to the prevention of miscarriages even not repeated (Giorlandino et al., 2009; Rubin, 2020), and a high prevalence of antibiotic use in the second semester of pregnancy for women age 35 and older, partly determined by antibiotic prophylaxis for invasive prenatal diagnosis, even if no clinical recommendations support this clinical practice (Gramellini et al., 2007; Benevent et al., 2019).

According to the ranking of the top therapeutic categories most prescribed in pregnant women, vitamins, minerals and antianemic preparations were the most frequently prescribed agents in combination with antibiotics for systemic use and progestins. Excluding the category of vitamins, minerals and antianemic preparations, the most prescribed drug combination was progestin with antibiotics for systemic use, the two therapeutic categories for which the most inappropriate use was detected during the study.

A consistent regional variability in medication prescription during pregnancy was detected, with a prevalence ranging from 65.7% of Lombardy to 86.1% of Sardinia; the drug categories with the highest variability in prevalence use were antianemic preparations and progestins.

A substantial heterogeneity in medication use during pregnancy was observed among specific population subgroups. Compared to Italian pregnant women, foreign pregnant women coming from high-income countries registered the lowest prevalence of drug prescriptions (61.5%), while women from low-income countries with large migration outflows had the highest one (74.9%). The drug prevalence in women with multiple pregnancies was higher (86.6%) than that observed in women with singleton pregnancy (72.9%), especially for progestins and medications containing folic acid.

## Discussion

MoM-Net represents one of the first Italian experiences in the creation of an infrastructure sharing regional health administrative data and coordinated by AIFA. The infrastructure interrogates and links different healthcare databases with an innovative approach converting the loco-regional data into a CDM, and exploits a virtuous model of collaboration between public institutions and researchers. This experience highlighted the importance of having a database network able to analyse real world data and describing demographic and clinical characteristics of patients exposed to specific medications, in order to provide rapid answers to urgent public health questions and promote evidence-based public health interventions (Toh et al., 2013).

The network created by AIFA investigating patterns of medication use before, during and after pregnancy is able to identify inappropriate or unsafe prescriptions. The first data analysis conducted by the network is an important novelty in the national panorama where the available data on drug prescription during pregnancy are still scarce and not updated (Gagne et al., 2008; Ventura et al., 2018). Moreover, the study findings provide a wealth of information that can be used in support of decision makers, health professionals (prescribers and pharmacists) and women of childbearing age or in pregnancy.

In recent years several countries have developed pharmacoepidemiology networks to answer specific research questions and used a distributed approach to preserve privacy, increase transparency, and reduce biases (EUROmediCAT, 2015; Andrade et al., 2016; Allotey et al., 2020). Studies using a distributed network approach and based on CDM have the advantage of being rapidly implementable at local level since they do not require specialised local centres, the analyses being run on the basis of scripts prepared by the coordinating centre whose task is also to investigate aggregate demographic, clinical, and prescription data of all centres participating in the network.

This approach minimizes data variability among regions, reduces program errors, is transparent because program scripts are shared within network and could be easily repeated in different contexts and over time in order to compare spatial and temporal trends in drug utilisation. Moreover, evidence and skills developed within the network will be useful to plan new studies and answer further clinical research questions.

Previous Italian network initiatives were part of funded pharmacovigilance projects and long-term stable networks able to perform pharmacoepidemiology studies are still lacking in the country. Recently AIFA has expressed interest in promoting and supporting such networks, in particular for studying the use of medicines in special populations whose information on drug utilization and safety may be lacking or very limited (Sultana et al., 2020). Knowledge gained from such experience will support both physicians in making well-informed treatment decisions and regulators in developing and implementing training and information activities. Further investigations are needed to analyse the impact of pregnancy on specific chronic diseases, assess the clinical effect of prescribing habits, evaluate prescribers’ attitude towards evidence-based guidelines and measure the impact of regulatory decisions on appropriate medication use in pregnancy.

In particular, information about chronic diseases, prescribers’ attitude and long-term baby outcomes could be extracted from administrative data available at regional level adding new data sources and variable of interest to our CDM and specific research hypotheses could be investigated. However, observational studies that will be conducted within MoM-Net could be affected by biases linked to data availability and quality. For example, the main limitations include the ability to monitor only drugs reimbursed by the Italian NHS and the lack of the indication for which the drug was prescribed. Anyway, nearly all drugs prescribed for chronic diseases (e.g. asthma, diabetes, epilepsy, hypertension) are reimbursed by the Italian NHS, and it is unlikely that such medications are payed out-of-pocket by patients. We are therefore confident to be able to adequately monitor drug therapies for the most common chronic diseases in pregnancy. Recently, the need for information has been more urgent since the outbreak of the new pandemic coronavirus disease (COVID-19) in the end of 2019. Evidence currently suggests that pregnant women with COVID-19 suffering from pre-existing chronic conditions, such as diabetes or cardiovascular diseases, as well as older and obese women are more likely to develop severe complications (Maraschini et al., 2020; Wastnedge et al., 2021). In addition, health care services reduced their ability to screen pregnant women for conditions such as gestational diabetes or hypertension, as well as make diagnosis and offer support for perinatal mental conditions, which could have, along with an increased socioeconomic deprivation, led to a wide range of adverse maternal and child health outcomes. A reduced access to reproductive healthcare services could be also expected (Zhao et al., 2020).

New research may help to increase understanding of the impact of COVID-19 on maternal and child health, in terms of direct effects of the infectious disease, indirect consequences of the pandemic on pre-existing chronic maternal drug therapy as well as in terms of the effects of medications used to treat the disease (i.e., corticosteroids, low-molecular-weight heparins, antiviral drugs), for which there is limited evidence on their use in pregnancy [39]. To answer these questions, the established MoM-Net could refer to data from a representative Italian maternal population, including a wide range of subgroups, particularly those at increased risk of worst outcomes.

The MoM-Net represents a potentially dynamic network able to guarantee a systematic surveillance on the use of medicines during pregnancy, monitor the prescriptions of drugs at high risk of inappropriateness, measure intra and interregional variability, and investigate health needs in specific population subgroups. Moreover, it may be used to support and evaluate specific interventions aimed to reduce inter and intraregional heterogeneity in drug prescriptions, and provide timely answers to any emerging questions on medication use during the perinatal period.

The potential extension of the MoM-Net in terms of participating Italian regions, institutions and data sources establishing a stable Italian network database, could favour the implementation of observational studies aimed to identify associations between drug exposure and pregnancy outcomes. A stronger alliance between the Italian regulatory agency, regional authorities and research institutions is essential to leverage Italian data more effectively for public health purposes.

## MoM-Net group

**Francesco Trotta**, **Filomena Fortinguerra**, **Serena Perna** (Italian Medicines Agency, Rome); **Valeria Belleudi**, **Francesca Romana Poggi**, **Antonio Addis**, **Marina Davoli**, (Department of Epidemiology, Lazio Regional Health Service, ASL Roma 1, Rome); **Serena Donati**, **Paola D’Aloja** (National Centre for Disease Prevention and Health Promotion, Istituto Superiore di Sanità, Rome); **Roberto Da Cas** (Pharmacoepidemiology Unit, National Centre for Drug Research and Evaluation, Istituto Superiore di Sanità, Rome); **Renata Bortolus**, **Giovanni Rezza** (Directorate General for Preventive Health - Office 9, Ministry of Health, Rome); **Antonio Clavenna** (Laboratory for Mother and Child Health, Department of Public Health - Istituto di Ricerche Farmacologiche Mario Negri IRCSS, Milan); **Anna Locatelli** (Obstetrics and Gynecology Unit, School of Medicine and Surgery, University of Milano Bicocca, Milan); **Arianna Mazzone**, **Simone Schiatti**, **Martina Zanforlini**, **Ida Fortino** (Lombardy Region); **Silvia Manea**, **Laura Salmaso**, **Giovanna Scroccaro**, **Paola Deambrosis** (Veneto Region); **Aurora Puccini**, **Valentina Solfrini**, **Anna Maria Marata** (Emilia-Romagna Region); **Rosa Gini**, **Francesco Attanasio** (Tuscany Region); **Marcello De Giorgi**, **David Franchini**, **Mariangela Rossi** (Umbria Region); **Lorella Lombardozzi** (Lazio Region); **Paolo Stella**, **Vito Bavaro**, **Vito Montanaro** (Apulia Region); **Stefano Ledda**, **Paolo Carta**, **Enrico Serra**, **Donatella Garau** (Sardinia Region).

## Data Availability

The data that support the findings of this study are available from the AIFA and are available from the authors upon reasonable request and with permission of the AIFA and partecipating Regions; Requests to access the datasets should be directed to v.belleudi@deplazio.it.
