# Supplementary material for: The Italian Network for Monitoring Medication Use During Pregnancy (MoM-Net): Experience and Perspectives
Source: Front Pharmacol. 2021 Jun 23;12:699062. doi: 10.3389/fphar.2021.699062 (PMC8262612; doi:10.3389/fphar.2021.699062)
Supplement: Supplementary file 1 [file Table1.DOCX]

Table 1. Prevalence of medicine use before, during and after pregnancy overall and according to: main therapeutic categories; Italian regions; Country of origin; Singleton/multiple pregnancy

|  |  | ***Three trimesters before***  ***pregnancy*** | ***Three trimesters during***  ***pregnancy*** | ***Three trimesters after***  ***pregnancy*** |
| --- | --- | --- | --- | --- |
|  |  |  |  |  |
| ***Overall*** | | 57.1 | 73.1 | 59.3 |
| ***Main therapeutic categories used in pregnancy*** | Vitamins and minerals | 2.3 | 2.9 | 2.3 |
|  | Antianemic preparations* | 10.4 | 34.6 | 4.9 |
|  | Treatment for acid-related disorders | 6.9 | 5.8 | 3.6 |
|  | Progestins | 5.4 | 20.2 | 0.4 |
|  | Gonadrotopins | 3.4 | 1.2 | 0.0 |
|  | Heparins | 2.0 | 5.2 | 22.6 |
|  | Antibiotics^#^ | 33.9 | 31.8 | 29.3 |
|  | Corticosteroids | 6.5 | 4.1 | 4.0 |
|  | Thyroid hormone | 4.3 | 7.7 | 5.0 |
|  | Anthypertensive^§^ | 1.2 | 2.0 | 2.9 |
|  | Antidiabetics° | 0.7 | 2.7 | 0.5 |
|  | Antiasthmatics | 8.3 | 6.9 | 5.9 |
|  | Antiepilectics | 0.7 | 0.4 | 0.6 |
|  | Psychotropics | 2.2 | 1.1 | 1.6 |
| ***Italian Regions*** | Lombardy | 52.1 | 65.7 | 54.2 |
|  | Veneto | 52.3 | 68.0 | 55.1 |
|  | Emilia Romagna | 58.6 | 78.8 | 61.7 |
|  | Tuscany | 56.5 | 71.4 | 59.2 |
|  | Umbria | 61.3 | 78.5 | 62.7 |
|  | Latium | 60.6 | 78.0 | 59.6 |
|  | Apulia | 66.4 | 83.3 | 70.3 |
|  | Sardinia | 67.3 | 86.1 | 72.7 |
| ***Women coming from*** | Italy | 58.6 | 72.8 | 60.3 |
|  | High-income countries | 48.1 | 61.5 | 51.1 |
|  | Low-income countries | 51.0 | 74.9 | 55.1 |
| ***Women with*** | Singleton pregnancy | 56.8 | 72.9 | 58.9 |
|  | Multiple pregnancy | 70.2 | 86.6 | 80.5 |

** Prevalence of folic acid use was: 10.4% before, 34.6% during and 4.9% after pregnancy*

*^#^ Prevalence of antibiotics by age class: ≤34 years: 33.1% before, 29.5% during and 39.1% after pregnancy; 35-39 years: 35.2% before, 34.8% during and 29.7% after pregnancy; ≥ 40 years: 35.4% before, 38.84% during and 29.7% after pregnancy.*

*^§^ Prevalence of calcium channel blockers use was: 0.3% before, 0.9% during and 1.4% after pregnancy; Prevalence of centrally acting antiadrenergic use was: 0.1% before, 0.7% during and 0.9% after pregnancy.*

*° Prevalence of insulins use was: 0.3% before, 2.4% during and 0.3% after pregnancy.*
